# High prevalence of obesity-related hypertension among adults aged 40 to 79 years in Southwest China

**DOI:** 10.1038/s41598-019-52132-6

**Published:** 2019-11-01

**Authors:** Yang Zhang, Li-Sha Hou, Wei-Wei Tang, Fan Xu, Rong-Hua Xu, Xin Liu, Ya Liu, Jian-Xiong Liu, Yan-Jing Yi, Tai-Shang Hu, Rong Hu, Tzung-Dau Wang, Xiao-Bo Huang

**Affiliations:** 1 Department of Cardiology, Second People’s Hospital of Chengdu, Chengdu, China; 2 Department of Geriatrics, West China Hospital, Sichuan University, Chengdu, China; 3 Department of Epidemiology and Health Statistics, West China School of Public Health of Sichuan University, Chengdu, China; 4 Public Health School, Chengdu Medical College, Chengdu, China; 5 Stroke Center, Second People’s Hospital of Chengdu, Chengdu, China; 6 Department of Epidemiology and Health Statistics, School of Public Health, Xi’an Jiao Tong University Health Science Center, Xi’an, Shanxi People’s Republic of China; 7 Department of Geriatrics, Second People’s Hospital of Chengdu, Chengdu, China; 8 Department of Epidemiology, Public Health and Management, Chongqing Medical University, Chongqing, China; 9 Division of Cardiology, The Second Affiliated Hospital of Chongqing Medical University, Chongqing, China; 10 Division of Cardiology, Department of Internal Medicine, National Taiwan University Hospital, Taipei City, Taiwan; 11 School of Health Policy and Management, Nanjing Medical University, Nanjing, 211166 Jiangsu Province P.R. China

**Keywords:** Cardiology, Hypertension

## Abstract

This study aimed to assess the prevalence and related factors of obesity-related hypertension among adults aged 40 to 79 years in Southwest China. From September 2013 to March 2014, a multi-stage, stratified sampling method was conducted on 10,589 people aged 40 to 79 years and living in Chengdu and Chongqing investigated by using a questionnaire and performing physical and biochemical measurements. The prevalence of obesity-related hypertension and hypertension overall (systolic ≥130 mmHg and/or diastolic ≥80 mmHg or treated hypertension) was 22.8% and 57.4%, respectively, among all participants. For obesity-related hypertension, the prevalence was higher in women than in men (24.7% versus 19.4%, *p* < 0.001). For people in the age ranges of 40–49, 50–59, 60–69, and ≥70, the prevalence of obesity-related hypertension were 11.8%, 22.6%, 30.7%, and 36.6%, respectively. Participants with obesity-related hypertension as opposed to those with non-obesity-related hypertension had a higher prevalence of hypertriglyceridemia, high low-density lipoprotein cholesterolemia, diabetes, and hyperuricemia (all *p* < 0.05). Multivariate logistic regression analysis showed that age, female gender, current smoking, hypertriglyceridemia, diabetes and family history of hypertension were all positively correlated with obesity-related hypertension, whereas higher education level and having spouse were negatively correlated with obesity-related hypertension. The prevalence of obesity-related hypertension was high among adults aged 40 to 79 years in Southwest China. Cardiometabolic abnormalities among participants with obesity-related hypertension were more serious and frequently present than in those with non-obesity-related hypertension. Aggressive and holistic strategies aiming at the prevention and treatment of obesity-related hypertension are needed.

## Introduction

Hypertension is a major risk factor that leads to myocardial infarction, stroke, renal failure, and death^1,2^. Obesity raises the risk of morbidity from hypertension, dyslipidemia, diabetes, coronary heart disease (CHD), stroke and heart failure^3–6^. Hypertension and obesity are both associated with increased risks of all-cause and cardiovascular mortality^7–9^, and they often occur together^10^. Socioeconomic and demographic transitions occurring in many developing countries have contributed to the burden of hypertension and obesity^2,11^, and the transition of morbidity from communicable diseases to non-communicable diseases^12,13^. With the rapid industrialization and urbanization seen in recent decades, the prevalence of hypertension and obesity has increased significantly in China, with the prevalence of obesity increased from 4.0% in 2002 to 10.7% in 2009^14^ and the prevalence of hypertension increasing from 18.8% in 2002^15^ to 29.6% in 2014^16^ in Chinese adults.

Obese patients with hypertension usually require more anti-hypertensive medications and have an increased risk of treatment-resistant hypertension^10^. In the past few years, the American Society of Hypertension, the Obesity Society, and the European Association for the Study of Obesity have published joint statements about obesity-related hypertension. However, little data has been published about the prevalence of obesity-related hypertension according to various world definitions.

Chengdu is one of the national centers for urban planning under the state council and is Southwest China’s science and technology, commercial, financial, traffic, and communication hub. Located in the upper Yangtze river region, Chongqing is one of the four municipalities directly under the central government and is also a commercial, financial, technical, innovative, and modern logistics center in Southwest China. In this study, we aimed to assess the prevalence of obesity-related hypertension in Southwest China, which is of value for the prevention and treatment of hypertension.

## Materials and Methods

### Study population

From September 2013 to March 2014, a multi-stage, stratified sampling was conducted on 10,589 people aged 40 to 79 years living in the urban communities of Chengdu and Chongqing investigated by using a questionnaire and performing physical and biochemical measurements. In the first phase of this study, the Jinjiang, Longquan, and Chenghua districts were randomly selected from the urban area of Chengdu; the Yubei and Jiangbei districts were randomly selected in Chongqing. In the second phase, a random sub-district was selected from each major district. In the third stage, one community was randomly selected from each sub-district, and a total of five random communities were selected. This study protocol was approved by the ethics committee of the Second People’s Hospital of Chengdu (NO 2013015), the methods in the study were in accordance with relevant guidelines, and a written informed consent was obtained from all participants.

### Inclusions and exclusions

Residents aged 40–79 years who had lived in the selected communities for more than five years were included in the study. People with a history of secondary hypertension, mental illness, malignancies, renal failure requiring dialysis, or who refused to participate in this inquiry were excluded. From September 2013 to March 2014, 11,385 people were invited to participate. Due to missing sex, blood pressures, weight, waist circumference (WC), or body-mass index (BMI) data, 796 patients were excluded. Thus, 10,589 patients were included in the final analysis.

### Data collection

More than 30 investigators were trained for data collection. All subjects filled out the same on-site questionnaire, which included demographic characteristics, lifestyle risk factors, and personal and family history, according to the cardiovascular survey methods of World Health Organization (WHO)^17^. The questionnaire also included height, weight, WC, and blood pressure measurements. When measuring the height and weight, the subject needed to be barefoot and take off any hat, wearing only lightweight clothes. The BMI was calculated by weight (kg) and by height (meters) squared. Investigators measured the minimum circumference between the inferior margin of the ribcage and the iliac crest as the WC measurement^18^. After the study, the subjects took a five-minute seated rest, then standardized mercury sphygmomanometers were used to measure their sitting blood pressures. Systolic blood pressure (SBP) and diastolic blood pressure (DBP) were recorded at the first appearance and disappearance of Korotkoff sounds. Two blood pressure readings were obtained and averaged.

### Blood sample collection and laboratory tests

Venous blood was drawn after 12 hours of fasting. Blood glucose, lipids, and uric acid (UA) levels were assessed in all blood samples. Patients without a history of diabetes mellitus (DM) were tested using the oral glucose tolerance test (OGTT), wherein 75 g of glucose was dissolved in 300 ml of warm water and was administered orally within five minutes, and venous blood was drawn two hours later. The total cholesterol (TC), triglycerides (TG), and blood glucose levels were detected by enzymatic methods. High-density lipoprotein cholesterol (HDL-C), low-density lipoprotein cholesterol (LDL-C) levels were measured using a homogeneous method. Serum UA was measured by the phosphotungstic acid method.

### Diagnostic standards

According to the recommendations from 2017 American College of Cardiology (ACC) Guidelines, hypertension was defined as an SBP ≥130 mmHg and/or DBP ≥80 mmHg, and/or being diagnosed with hypertension and currently under antihypertensive drug treatment^19^. DM was defined as a fasting plasma glucose (FPG) level ≥7.0 mmol/L, 2-hour postprandial glucose (2-hPG) level ≥11.1 mmol/L, or a previous clinical diagnosis^20^. Overweight was defined as a BMI between 24.0 and 27.9 kg/m^2^. Obesity was defined as a BMI of 28.0 kg/m^2^ or more. Central obesity was defined as a WC of 90 cm or more in men and 85 cm or more in women^21,22^. According to the consensus of Chinese experts on obesity-related hypertension management and ACC guidelines, obesity-related hypertension was defined as a BMI of 28.0 kg/m^2^ or more and/or a WC of 90 cm or more in men and 85 cm or more in women, SBP ≥130 mmHg and/or DBP ≥80 mmHg, and/or being diagnosed with hypertension and currently under antihypertensive drug treatment. Participants with secondary hypertension were excluded from this study^23^. Hypertriglyceridemia was defined as a TG level ≥1.7 mmol/L. High LDL cholesterolemia was defined as a LDL-C level ≥3.4 mmol/L. Hypercholesterolemia was defined as a TC level ≥5.2 mmol/L based on the criteria of the NCEP Adult Treatment Panel III report^24^. A history of smoking was defined as smoking at least once per day for more than a year, and currently having smoked or quit smoking for less than three years. A history of drinking was defined as drinking at least once a week over a year, and currently having drunk or quit drinking for less than three years. The family history of hypertension was defined as immediate family members having hypertension. Physical exercise was defined as having at least one exercise session per week.

### Statistical analysis

The Epidata3.1 software was used to double the input data to ensure their quality, and data processing and analysis were carried out using the SAS 9.2 software (Institute Inc. SAS, Cary, NC, USA). Quantitative data were compared using the one sample t-test or the Wilcoxon rank sum test, and qualitative data were compared using the χ^2^ test. The χ^2^ linear trend test was used to detect the trend in the prevalence of obesity-related hypertension in individuals in association with their age and BMI. Logistic regression was used to explore the potential risk factors for obesity-related hypertension. The covariates selected in the logistic regression model included age, sex, having or without spouse, education level, income, smoking, drinking, like to eat fried food (more than 3 times a week),family history of hypertension, daily main food more than 300 g/d or not, physical exercises, diabetes, hypertriglyceridemia, hypercholesterolemia, and hyperuricemia. A *p*-value of <0.05 was considered as significant.

## Results

### Prevalence of hypertension and obesity-related hypertension

**Figure 1:**
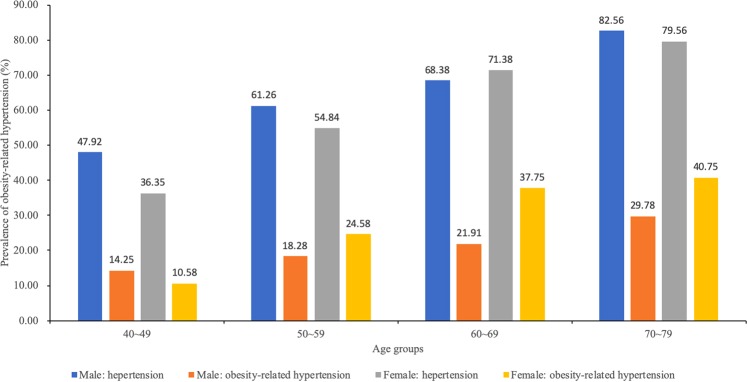
The prevalence of hypertension and obesity-related hypertension across different age groups.

In this study, 10,589 adult participants aged 40 to 79 years in Southwest China were included, among whom 3,823 were men and 6,766 were women. The overall prevalence of hypertension was 57.4% (6,083/10,589), with obesity-related hypertension accounting for 39.6% (2,410/6,083) of hypertension cases and 22.8% (2,410/10,589) of all participants. Obesity-related hypertension was more common in women than in men (24.7% versus 19.4%, *p* < 0.001). In men, obesity-related hypertension accounted for 31.4% (742/2,365) of hypertensive patients, while in women, it accounted for 44.9% (1,668/3,718) of the hypertensive patients. There were differences in age, sex, marriage, and education between the four groups stratified by the presence or absence of obesity and hypertension (Table 1). The prevalence of hypertension and obesity-related hypertension increased with age (*p* < 0.001) in both sexes (Fig. 1). At age 70 and above, nearly 30% of men and 40% of women suffered from obesity-related hypertension. Participants without spouse had a higher prevalence of obesity-related hypertension (*p* < 0.001). The participants with lower education levels had a higher prevalence of obesity-related hypertension (*p* < 0.0001). There were no significant differences in the prevalence of obesity-related hypertension between the high-income and low-income groups.

**Table 1:** Baseline characteristics of the study participants.

Characteristics	Normal population (n = 3598)	Obesity without hypertension (n = 908)	Non-obesity-related hypertension (n = 3673)	Obesity-related hypertension (n = 2410)	*P* values
Sex					<0.0001
Male	1227(34.1)	231(25.4)	1623(44.2)	742(30.8)	
Female	2371(65.9)	677(74.6)	2050(55.8)	1668(69.2)	
Age, years					<0.0001
40~49	1370(38.1)	213(23.5)	753(20.5)	313(13.0)	
50~59	1389(38.6)	411(45.3)	1411(38.4)	926(38.4)	
60~69	548(15.2)	186(20.5)	964(26.2)	752(31.2)	
≥70	127(3.5)	82(9.0)	478(13.0)	396(16.4)	
Spouse					<0.0001
No	255(7.1)	72(7.9)	342(9.3)	257(10.7)	
Yes	3318(92.2)	825(90.9)	3313(90.2)	2141(88.8)	
Income					0.26
≤2000 yuan	2825(78.5)	716(78.9)	2937(80.0)	1906(79.1)	
>2000 yuan	730(20.3)	186(20.5)	679(18.5)	468(19.4)	
Education					<0.0001
middle school or below	2510(69.8)	709(78.1)	2769(75.4)	2009(83.4)	
High school education or above	1073(29.8)	196(21.6)	875(23.8)	388(16.1)	

Values are presented as number (%).

### Characteristics of the obesity-related hypertensive and non-obesity-related hypertensive patients

The obesity-related hypertensive patients were older and had a higher BMI, WC, Hip circumference, SBP, TG, FPG, 2h-PG, UA, and LDL-C(all *p* < 0.05) and lower HDL-C (*p* < 0.05)(Table 2). There were no differences in height, DBP, heart rate (HR), or TC.

**Table 2:** Baseline characteristics of the obesity-related hypertensive and non-obesity-related hypertensive patients.

Variable	Overall	Non-obesity-related hypertension (N = 3673)				Obesity-related hypertension (N = 2410)				*P* ^*†*^
		Overall	Men (N = 1623)	Women (N = 2050)	P*	Overall	Men (N = 742)	Women (N = 1668)	P*	
Age, years	57.81(29.43)	58.72(18.90)	59.11(10.18)	58.41(23.62)	0.231	62.52(38.29)	62.74(42.1)	62.42(36.48)	0.859	<0.001
Height, cm	157.3(11.89)	157.02(8.50)	163.08(6.19)	152.23(6.85)	<0.001	157.13(8.72)	165.18(7.47)	153.54(6.57)	<0.001	0.638
Weight, kg	59.17(13.88)	56.32(8.03)	60.7(7.42)	52.85(6.68)	<0.001	67.21(9.84)	74.1(8.53)	64.14(8.77)	<0.001	<0.001
BMI, kg/m^2^	23.83(3.42)	22.78(2.38)	22.81(2.34)	22.76(2.41)	0.543	27.14(3.10)	27.11(2.68)	27.16(3.27)	0.716	<0.001
waist circumference, cm	81.56(25.06)	77.46(7.24)	79.95(7.16)	75.49(6.67)	<0.001	92.81(6.27)	95.07(5.70)	91.8(6.25)	<0.001	<0.001
Hip circumference, cm	93.62(13.74)	91.37(14.71)	91.68(6.38)	91.13(18.84)	0.216	100.37(7.47)	100.44(6.41)	100.34(7.90)	0.744	<0.001
systolic pressure, mmHg	130.72(21.38)	142.1(18.31)	142.22(18.45)	142.01(18.2)	0.733	145.49(19.23)	144.98(18.14)	145.72(19.70)	0.371	<0.001
Diastolic pressure, mmHg	79.14(18.6)	85.59(18.28)	86(12.28)	85.26(21.89)	0.197	86.43(25.97)	87.84(20.83)	85.81(27.94)	0.048	0.167
Heart rate/min	79.99(25.84)	81.32(20.04)	80.98(26.69)	81.58(12.51)	0.410	82.45(36.44)	82.86(44.40)	82.27(32.29)	0.744	0.166
TC, mmol/L	4.63(0.93)	4.69(0.94)	4.57(0.90)	4.79(0.97)	<0.001	4.76(0.91)	4.59(0.88)	4.84(0.91)	<0.001	0.05
HDL-C, mmol/L	1.42(0.36)	1.43(0.32)	1.38(0.34)	1.47(0.30)	<0.001	1.33(0.31)	1.24(0.29)	1.37(0.31)	<0.001	<0.001
LDL-C, mmol/L	2.52(0.76)	2.55(0.77)	2.48(0.77)	2.61(0.77)	<0.001	2.63(0.77)	2.6(0.79)	2.65(0.76)	0.166	<0.001
TG, mmol/L	1.62(1.25)	1.57(1.24)	1.61(1.30)	1.55(1.19)	0.179	2.06(1.47)	2.01(1.35)	2.08(1.51)	0.245	<0.001
FPG, mmol/l	5.69(2.07)	5.72(1.89)	5.8(2.05)	5.6 (1.75)	0.02	6.14(2.34)	5.94(1.77)	6.23(2.54)	0.001	<0.001
2hPG, mmol/L	7.91(3.83)	7.99(3.69)	7.88(3.87)	8.08(3.54)	0.131	9.29(4.46)	8.71(3.63)	9.56(4.77)	<0.001	<0.001
Uric acid, mmol/L	291.8(83.26)	293.59(83.96)	336.54(86.12)	259.87(64.62)	<0.001	315.26(89.47)	376.5(86.96)	288.4(76.4)	<0.001	<0.001

Values are presented as mean (SD).

*p for comparison between men and women within either obesity-related hypertension or non-obesity-related hypertension.

†*p* for comparison between overall obesity-related hypertensive patients and non-obesity-related hypertensive patients.

### Univariate analyses

Differences in the prevalence of cardiometabolic risk factors in obesity-related hypertensive and non-obese-related hypertensive subgroups are presented in Table 3. The obesity-related hypertensive patients had a higher prevalence of hypertriglyceridemia, diabetes, hyperuricemia and higher LDL-C (all *p* < 0.05). There was no difference in the prevalence of hypercholesterolemia (*p* = 0.60).

**Table 3:** Prevalence of cardiovascular risk factors in the obesity-related hypertensive and non-obesity-related hypertensive patients.

Variable	Overall	Non-obesity-related hypertension (N = 3673)				Obesity-related hypertension (N = 2410)				*P* ^*†*^
		Overall	Men (N = 1623)	Women (N = 2050)	P*	Overall	Men (N = 742)	Women (N = 1668)	P*	
Hypertriglyceridemia	2207(36.3)	1041(28.3)	494(30.4)	547(26.7)	0.012	1166(48.4)	352(47.4)	814(48.8)	0.537	<0.001
Hypercholesteremia	788(13.0)	469(12.8)	154(9.5)	315(15.4)	<0.001	319(13.2)	59(8.0)	260(15.6)	<0.001	0.595
High low-density lipoprotein cholesterolemia	756(12.4)	427(11.6)	142(8.7)	285(13.9)	<0.001	329(13.7)	90(12.1)	239(14.3)	0.147	0.019
Diabetes mellitus	1635(26.9)	808(22.0)	362(22.3)	446(21.8)	0.690	827(34.3)	228(30.7)	599(35.9)	0.013	<0.001
Hyperuricemia	799(13.1)	347(9.4)	217(13.4)	130(6.3)	<0.001	452(18.8)	200(26.9)	252(15.1)	0.022	<0.001

Values are presented as number (%).

*p for comparison between men and women within either obesity-related hypertension or non-obesity-related hypertension.

^†^*p* for comparison overall obesity-related hypertensive patients and non-obesity-related hypertensive patients.

### Multiple logistic regression

Multiple logistic regression analysis was used to determine the factors associated with obesity-related hypertension. The analysis showed that aging, current smoking, female gender, family history of hypertension, hypertriglyceridemia and diabetes were positively correlated with obesity-related hypertension. Higher education level and having spouse were negatively correlated with it. The results are presented in Table 4.

**Table 4:** Multivariable-adjusted odds ratios for obesity-related hypertension.

Characteristics	Odds Ratios (95% CI)	*P*
Female	1.29 (1.03,1.62)	0.025
Age	2.37 (1.63,3.23)	<0.0001
High school education or above	0.46(0.37, 0.57)	<0.0001
Current smoking	1.30(0.78, 1.98)	<0.0001
Family history of hypertension	1.29 (1.07, 1.56)	0.008
Hypertriglyceridemia	1.68(1.05, 2.42)	<0.0001
Diabetes mellitus	1.80 (1.27, 2.43)	<0.0001
Having spouse	0.76 (0.58, 0.95)	0.006
Hyperuricemia	2.01 (1.49, 2.69)	<0.0001

Age, sex, having or without spouse, education level, income, smoking, drinking, like to eat fried food (more than 3 times a week), family history of hypertension, family history of diabetes, daily main food more than 300 g/d or not, physical exercises, diabetes, hypertriglyceridemia, hypercholesterolemia, and hyperuricemia were treated as independent variables in the multifactorial logistic regression model.

## Discussion

This is the first report about the prevalence of obesity-related hypertension among Chinese adults according to definitions set by 2017 ACC Guidelines, which is one of the study strong points. In this population-based investigation, we found that the prevalence of obesity-related hypertension and hypertension overall were 22.8% and 57.4%, respectively, among adults aged 40 to 79 years in Southwest China. This finding indicates that nearly 40% of hypertensive patients had obesity-related hypertension and that nearly 1 in 4 adults aged 40 to 79 years had obesity-related hypertension. Given the approximately 10% prevalence rate of obesity in China, the prevalence of obesity (~30%) and obesity-related hypertension (22.8%) were high among adults aged 40 to 79 years in Southwest China.

We showed that the prevalence of hypertension was higher in men than in women, similar to the previous studies^15,16^. In contrast, obesity-related hypertension was more prevalent in women (24.7%) than in men (19.4%). In this study, about 1/3 of men suffering from hypertension had obesity-related hypertension, whereas approximately 1/2 of women suffering from hypertension had it. The higher prevalence of obesity-related hypertension in women is partly due to the fact that women had nearly 1.4 times higher prevalence of obesity than men (34.7% versus 25.5%). Moreover, our study found that the prevalence of hypertension in obese participants was significantly higher than in non-obese participants (72.6% versus 50.5%). A prior study was done in 14 provinces of China also showed that the prevalence of obesity in women was higher than in men and the prevalence of hypertension was increased with BMI^25^, consistent with our observations. Other studies were done in China besides Southwest region have also shown a positive correlation between the prevalence of hypertension and BMI^26,27^. In order to achieve better control of hypertension in a community perspective, it is necessary to check blood pressures regularly in obese patients, especially those who are severely obese.

Some studies reported that a higher education level was associated with lower prevalence of hypertension^28–30^and that there was an inverse relationship between the educational level and obesity^31–33^. In our investigation, a higher education level was associated with a lower prevalence of obesity-related hypertension, while there were no significant differences between the high-income and the low-income groups. It has been suggested that in a period of economic development, it is of great importance to improve the population’s level of cultural knowledge, especially in terms of health, to improve the health of the population as a whole. Our study showed that unmarried people had a higher prevalence of both hypertension and obesity-related hypertension. Former studies reported that unmarried persons were positively correlated with hypertension and had an increased mortality from cerebrovascular diseases^34–36^. Previous research assumed that married people could improve their quality of life and medication compliance in geriatric patients with chronic illnesses^37–39^. We speculate that most of the middle-aged and elderly who have a spouse are emotionally more stable and eat more regularly, though further study is needed.

Previous studies have suggested that smoking leads to hypertension^40,41^, mainly because it stimulates the sympathetic nervous system and accelerates atherosclerosis^42^. Previous studies have also suggested that smoking leads to a decline in BMI and the prevalence of obesity^43,44^. One study suggested that cigarette smoking was positively associated with abdominal obesity^45^. Our study suggested that smoking may increase the prevalence of obesity-related hypertension and is a potential risk factor for it, which also requires further study.

A higher prevalence of hypertriglyceridemia, DM, and hyperuricemia was observed in the obesity-related hypertension group, which has been ascribed to be related to central obesity, insulin resistance, and subclinical inflammation^46–49^. Besides, osteoprotegerin, camellia sinensis extract, mineral metabolism, has been ascribed to be related to arteriosclerosis, insulin resistance, and lipids metabolism in hypertentsion^50–53^. In this study, approximately half of obesity-related hypertensive patients had hypertriglyceridemia and there were 1 diabetic patients in every 3 obesity-related hypertension patients, aged 40 to 79 years. Hypertension, obesity, and DM have become the leading causes of cardiovascular disease and death in many developing countries^54–56^, and a higher UA level has been associated with an increased risk of hypertension^57,58^. In addition, we found that the prevalence of high LDL-C was higher in obesity-related hypertensive patients. Compared to the non-obesity-related hypertensive patients, regular screenings for cardiovascular metabolic risk factors in obesity-related hypertensive patients should be advocated.

The strengths of this study include a large number of community-dwelling participants included in this survey and a detailed report of hypertension and obesity-related hypertension based on the 2017 ACC Guidelines in Southwest China. Our study has several limitations. First, this study was a cross-sectional study, meaning that the findings cannot be used to establish a conclusive cause-and-effect relationship between risk factors and obesity-related hypertension. Second, the participants were recruited from Southwest China, such that the conclusions cannot represent the situation in other regions of China. Third, we did not perform detailed body composition analysis due to constraints of budgets.

## Conclusions

The prevalence of obesity-related hypertension was high in people aged 40 to 79 years in Southwest China. Its prevalence was higher in women than in men and increased with age. Additionally, patients with obesity-related hypertension had a higher prevalence of DM, hypertriglyceridemia, high low-density lipoprotein cholesterolemia and hyperuricemia than non-obesity-related hypertensive patients. Aggressive strategies aiming at the prevention and treatment of obesity-related hypertension are needed.
